# Supplementary figures and images for: Genome-Wide Identification, Evolution and Expression Analysis of GRAS Transcription Factor Gene Family Under Viral Stress in Nicotiana benthamiana
Source: Plants (Basel). 2025 Jul 25;14(15):2295. doi: 10.3390/plants14152295 (PMC12348890; doi:10.3390/plants14152295)

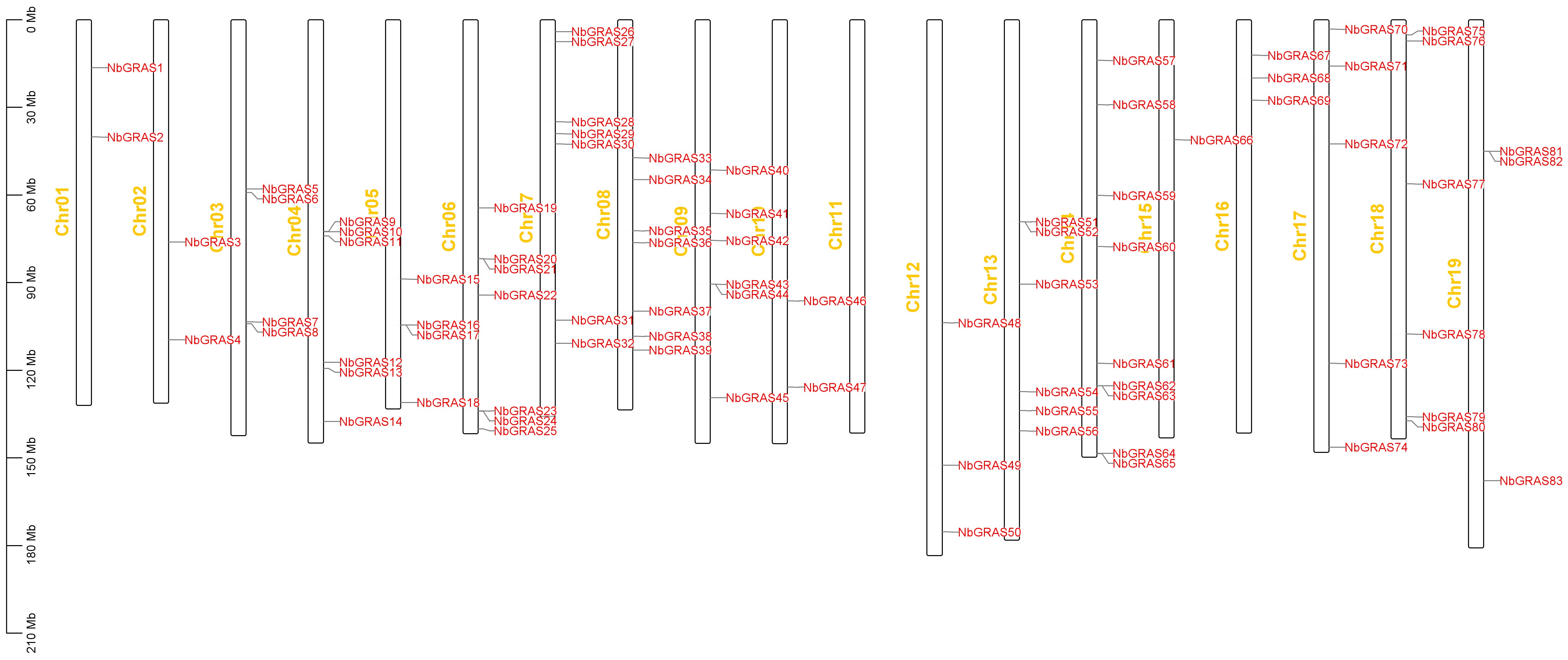

Supplement: Supplementary file 1 [file plants-14-02295-s001.zip › Fig S1.jpg]

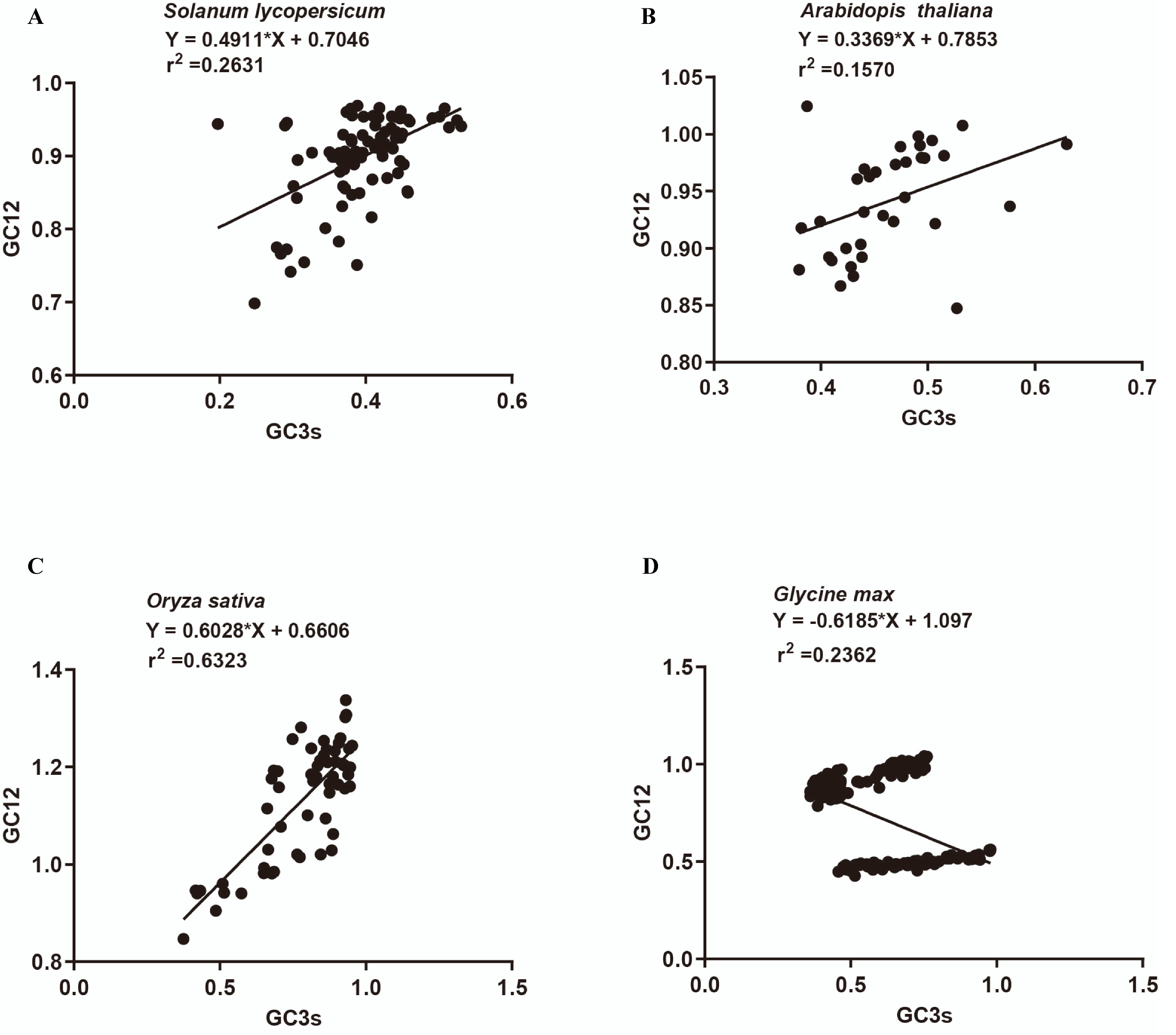

Supplement: Supplementary file 1 [file plants-14-02295-s001.zip › Fig S2.png]

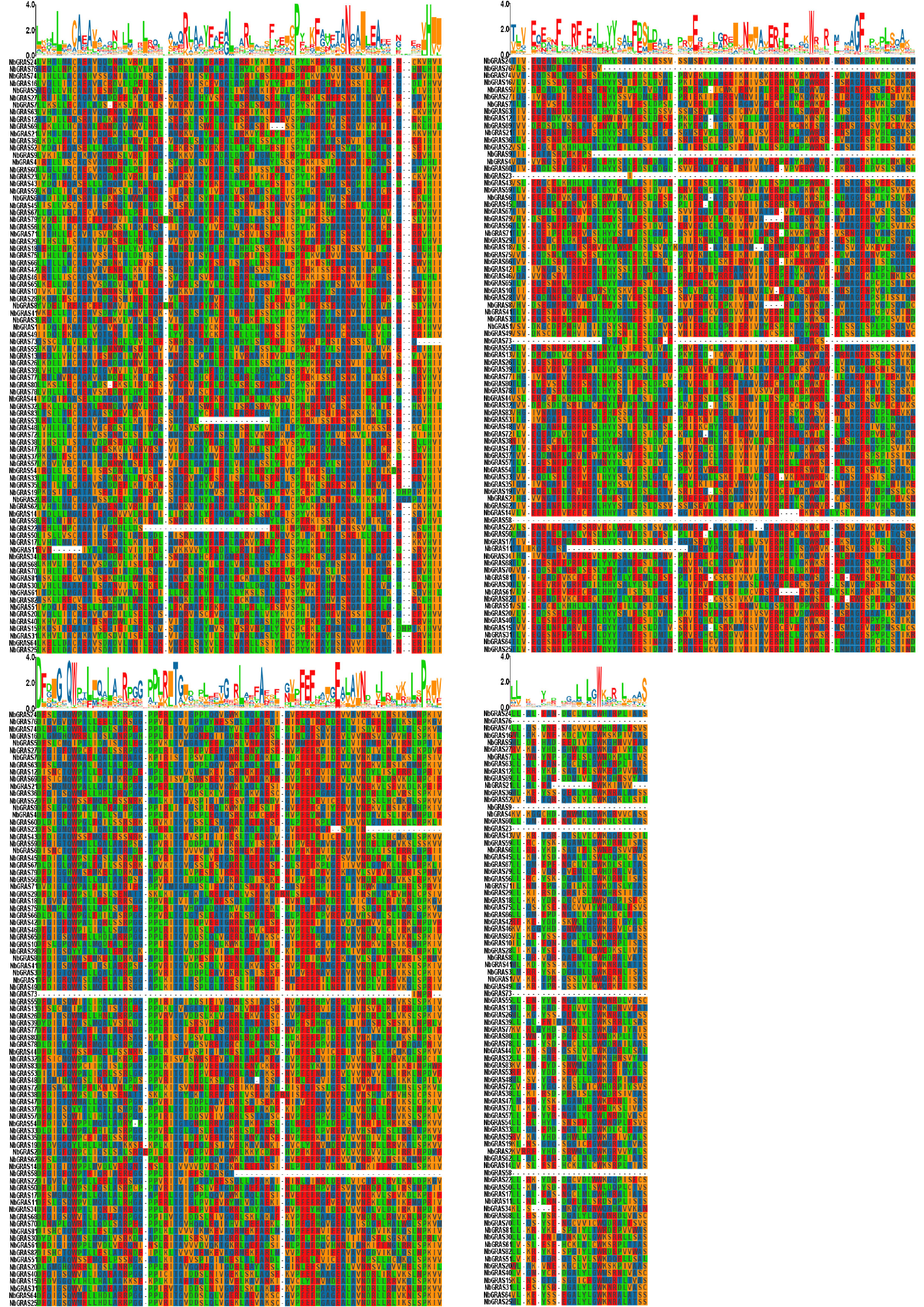

Supplement: Supplementary file 1 [file plants-14-02295-s001.zip › Fig S3.jpg]
